# Framework for Risk Communication and Community Engagement to Improve Vaccine Uptake for COVID-19 and other Vaccine-Preventable Diseases in Low- and Middle-Income Countries (LMIC)

**DOI:** 10.5334/aogh.3483

**Published:** 2021-12-09

**Authors:** Laston Gonah, Aminata Grace Kobie

**Affiliations:** 1 Department of Community Medicine, Midlands State University, Zimbabwe; 2 World Health Organization Office for Africa, Congo

## Abstract

A framework for guiding risk communication and community engagement (RCCE) during COVID-19 vaccination roll-out is worthwhile in order to guide interventions aimed at improving vaccine uptake. This requires setting up standardised early-warning indicators to predict or detect low uptake; coordination of response activities by all partners, real-time information exchange, innovativeness in designing strategies to deal with arising and anticipated challenges; flexibility to adapt quickly to changing demands and evolving circumstances; and documentation of progress and lessons learnt.

## Background

Most countries across the world have already started COVID-19 vaccine roll-out programmes, using one or more types of COVID-19 vaccines [1] such as Pfizer, Johnson & Johnson, AstraZeneca, Sinovac, and Moderna. The type(s) of vaccine(s) used varies from country to country and from time to time as influenced by various factors such as international and national vaccine regulations and approvals, reported vaccine effectiveness, available vaccine financing options, vaccine transportation and storage requirements, logistics and costs associated with vaccine procurement and roll-out, among others.

Vaccination uptake at the start of the roll-out has been shown to vary between continents, among countries and within countries for various reasons. In most low- and middle-income countries (LMICs), COVID-19 vaccine uptake is not yet optimum despite the countries having received vaccine allocations from various sources. Noting the general vaccine hesitancy; lower COVID-19 risk perception than the actual risk, together with low optimum uptake of COVID-19 vaccines, there is need to develop a framework for guiding crisis communication to better coordinate and monitor issues of vaccination that need communication support.

## Framework objectives

1. To define standardized early-warning indicators/signs for low vaccine uptake and vaccine hesitancy
  – Adverse events following immunization (AEFIs), at least one of those outlined by World Health Organization (WHO) [2] or Centers for Disease Control and Prevention (CDC) [3], or any other relevant body
  – Persistent misinformation and disinformation
  – Risk perception lower than actual risk
  – Vaccine shopping
  – Risk of vaccine expiration
  – Funding challenges to implement vaccination or vaccine communication programmes
  – Vaccine usage below set targets
  – Vaccine shortages
2. To outline an implementation progress checklist for key crisis communication activities
  – Consistent national social listening team(s) and rapid surveys
  – Engage vaccine champions, trusted community channels and leaders
  – Listen and communicate widely, and clarify the process (explain how vaccines work—benefits and common adverse events, remind the public about vaccination schedule)
  – Build trust by sharing daily accurate updates on vaccine rollout
  – Publicise the correct number of people already vaccinated, and those intending to, where possible
  – Make better use of local TV and radio stations, and other official social media platforms
  – Develop and circulate correct, contextualized and appropriate COVID-19 information, education and communication (IEC) materials in all languages or info grams in situations where illiteracy is an issue (based on identified information needs); and offer training to outlets/partners on usage
  – Ensure sufficient coverage of interventions in all geographical areas at no form of cost to individuals intending to take the vaccine, such as missed work opportunities, management of adverse events following immunization (AEFIs), transport costs
3. To define the best approach for harmonizing and coordinating crisis communication activities by all partners in a defined country or geographical region/area
  – Regular feedback meetings/webinars/refresher trainings
  – Alignment and harmonization of plans, strategies and approaches
  – Engagement with government ministries, Department or Ministries of Health, and so on
  – Platforms for information rapid exchange groups like WhatsApp, Signal, and so on
  – Platforms for real-time early signs data access

### Framework for improving vaccine uptake

**Figure:**
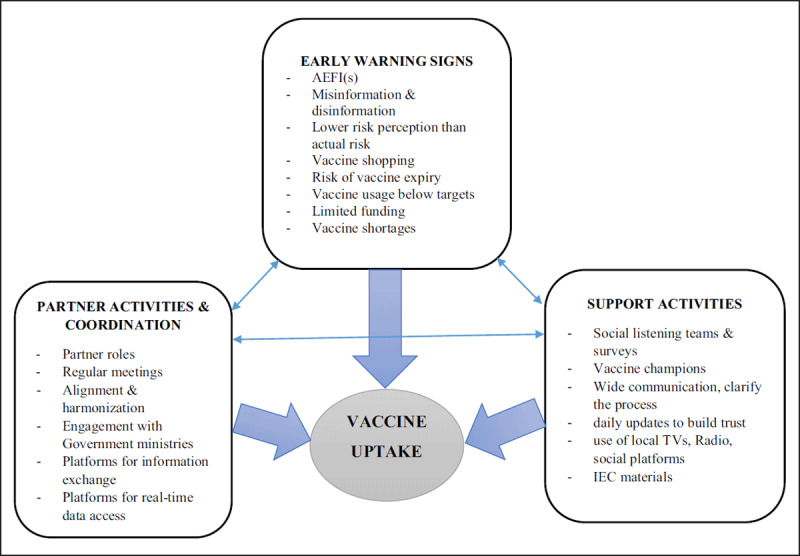
*N.B: There may be other elements in addition to those shown in the above framework*.

## Explanation

Improving vaccine uptake requires consideration of, and attention to, various aspects of early warning signs, vaccine roll-out support activities, as well as partner activities and coordination for vaccine roll-out. The factors or elements shown in the framework are inextricably linked together in producing observed vaccine uptake status or outcome. Therefore, there is no one aspect or factor which is more important than the other in influencing vaccine uptake by the target population, rather the factors are interlinked and interdepended on each other in producing the observed vaccine uptake status.

### Framework for improving vaccine uptake

**Table:** 


FRAMEWORK ELEMENTS	STANDARD THRESHOLDS FOR ACTION/RESPONSE ACTIONS	GOALS

**Early warning signs** – AEFIs (at least one) – Persistent misinformation and disinformation – Risk perception lower than the actual risk – Risk of vaccine expiration – Limited funding to implement support activities – Vaccine usage below set targets – Vaccine shopping	– Be the first to explain any AEFIs – Provide accurate information – Increase acceptance and demand for vaccination – Explore all available funding sources – Troubleshoot to identify challenges and intervene – Advocate against vaccine shopping	– Address fears – Reduced vaccine hesitancy – improved knowledge on vaccines – Improved vaccine uptake – Mobilise funding to support demand creation/RCCE – Be ahead of time – Avoid vaccine shopping

**Support activities** – Consistent national social listening teams and surveys – Engage vaccine champions, trusted community channels and leaders – Communicate widely and clarify the process – Build trust by providing accurate daily updates on vaccine rollout – Make better use of local TV and radio stations, and other official social media platforms – Develop and circulate COVID-19 IEC materials in all languages (based on identified information needs); and offer training to outlets/partners on usage	– Collect feedback from hotlines, AEFI monitoring tool, traditional and social media on regular basis – Empower local leadership and trusted community channels and leaders with accurate information – Regularly collect feedback and update messages	– Up-to-date information on hesitancy – Easy-to-understand information – Continued risk perception (appropriate to the actual risk and increased demand for vaccination) – Community participation and ownership

**Partner activities and collaboration** – Regular feedback meetings/webinars/refresher trainings – Alignment and harmonization of plans, strategies and approaches – Engagement with government ministries, Department or Ministry of Health, and so on – Platforms for information exchange groups like WhatsApp, Signal, and so on – Platforms for real-time early signs data access	– Stakeholder mapping – Set up committees – Maintain stakeholder presence and participation – Agree on stakeholder roles – Share feedback and discuss progress (results) – Agree on frequency of meetings – Refresher trainings – Ensure real-time data access and encourage partners to consistently update information – Revise approaches and strategies	– Harmonised approach – Coordinated response – Consistency (avoid parallel programs and conflicting messages/interventions) [4] – Fast and efficient information exchange – Reliable data/information and timely response – Innovative and effective approaches – Mobilised funds


## Limitations

The framework can only be used in guiding interventions and activities to improve vaccine uptake, not as an evaluating tool to assess progress. Countries and organizations need to develop thresholds or standardised indicators to determine various levels of crisis situations and to measure impact of their interventions, as may be applicable to a defined context.

## Conclusions

Improving vaccine uptake requires setting up early warning indicators to timely predict or detect low uptake. This requires coordination of response activities by all partners involved, real-time information exchange, innovativeness in designing strategies to deal with arising and anticipated challenges, flexibility to adapt quickly to changing demands and evolving circumstances, and documentation of progress and lessons learnt.
